# From Manila, P. I.

**Published:** 1901-09

**Authors:** 


### COMMUNICATION.

### FROM MANILA, P. I.

Dr. W. W. Richards, City Veterinarian of Manila, Philippine Islands, sends the following notes:

The native horse of these islands is a degenerate race, the males being left entire, with the consequent inbreeding, although, to say the least, he is possessed of wonderful vitality. They range from forty-six to fifty-two inches in height, and are chiefly used for transportation purposes.

Glanders is very prevalent among the native horses, but a great many of them have been policed up since American occupation. We test quite a number every month, under the direction of the Board of Health, and I may say a good word for "mallein," to the effect that in every case that a reaction has resulted an autopsy reveals the presence of "glanderous lesions."

The cattle industry of these islands has been virtually ruined within the last three years owing to the dread disease "rinderpest." Enclosed, you will find circular containing instructions in English and Spanish, which literature has been widely distributed among all the islands where American troops are stationed. These instructions are formulated from the Koch experimentation of immunization in South Africa.

Lieutenant Calvert, Assistant Surgeon, U. S. A., who is detailed as bacteriologist to the Manila Board of Health, has been making extensive investigations in regard to rinderpest in some of the affected districts on the southern portion of the Island of Luzon, an account of which will soon be placed in print.

About one month ago one shipment of eighty-six head from an infected district was embarked for the port of Manila, eleven head dying en voyage of sixty hours. On inspection before unloading I detected symptoms of the disease, and ordered them unloaded in a casco, which was towed up the Pasig River to our quarantine station, landing fifty-one live animals. These were injected immediately with 50 c.c. of a glycerinated preparation of bile taken from those which were so far gone with the disease and destroyed by me aboard the vessel. The bile was injected after being in an ice-cooler some sixteen hours. Of the fifty-one head landed in quarantine, ten of them are undoubtedly immune. Remember, we were aware of the fact that no new disease was being introduced into the City of Manila, but quarantined this shipment for experimentation purposes, and now we are possessed of a source from which to draw serum.

At the present time these animals are pasturing on a hacienda about three miles from the City of Manila, where, about three months ago, one fine herd of milch cows, containing some fifty head, brought here from Australia, died after being placed on this hacienda some six weeks before.

There is a great field for experimentation with rinderpest on these islands, and our Government is surely in duty bound forced to take action immediately to endeavor to eradicate this disease, or at least as soon as the state of unrest among the natives justifies such steps being taken.

MUNICIPAL LABORATORY, OFFICE OF THE BOARD OF HEALTH, CIRCULAR LETTER No. 6,
MANILA, P. I., June 10, 1901.

*Rinderpest.* The importance of this disease to the welfare of the Philippines renders imperative the publication of knowledge gained from most recent investigation. This circular should be considered a supplement to Circular Letter No. 4, March 12, 1901, office of the Board of Health, Manila, P. I., on the same subject, and is distributed because of additional information acquired during the recent epidemic of rinderpest near Manila. The period of incubation of this pest is short, varying from three or four to eight days. It is necessary that cattle owners recognize the disease early in order to take steps to prevent its spread. Fever is the first symptom observed. Nothing else wrong may be noticed for two or three days, then the symptoms as noted in the above-mentioned circular become prominent. In addition great prostration, with evident intense suffering of animals from abdominal pain, is characteristic. Later in the disease the watery discharge from eyes and nose becomes purulent, frequently occluding the nares. The duration of the pest from the first symptom to death is two to five or six days.

No other disease affecting cattle can be mistaken for rinderpest, as will be seen by a recapitulation of its symptoms: loss of appetite, high fever, inflammation of conjunctivæ, profuse watery discharge from eyes and nose, finally becoming purulent; diarrhœa, often bloody; great prostration, and apparently intense abdominal pain; high mortality. The symptoms are practically given in the order of their occurrence, although the diarrhœa may occur very early.

The following methods of procedure will check the spreading of the disease in a community, but offers little encouragement for the treatment of animals already sick :

1. Remove the gall-bladder of all animals dead of rinderpest, taking as little of the liver substance as possible. Wash the bladder in a 2 per cent. carbolic-acid solution, then several times in water which has been boiled and allowed to cool ; now draw off the gall through the gall-duct by a puncture through the gall-bladder. A bottle boiled and allowed to cool should be used for this gall, which must be mixed with glycerin in the proportion of 1 to 3. The gall and glycerin should be kept in a cool place for four days, after which period it may be used. The mixture will keep for four months. Care must be taken not to use any bile of a decided yellow color.

2. Clean and disinfect in a 5 per cent. carbolic-acid solution the syringe for injecting the fluid. Leave the syringe in carbolic acid for ten minutes, then rinse in boiled water several times in order to remove the carbolic acid. Wash the hide of the animal at the point of inoculation with water and soap, then with a 5 per cent. solution of carbolic acid. Inject 50 c.c. beneath the skin of the belly wall of all the animals of the herd. Reinject in from eight to ten days after first injection, using 100 c.c.

3. Treat herd as directed in Circular Letter No. 4, office of the Board of Health, Manila, P. I., March 12, 1901.

